# The Two-Step Clustering Approach for Metastable States Learning

**DOI:** 10.3390/ijms22126576

**Published:** 2021-06-19

**Authors:** Hangjin Jiang, Xiaodan Fan

**Affiliations:** 1 Center for Data Science, Zhejiang University, Hangzhou 310058, China; jianghj@zju.edu.cn; 2 Department of Statistics, The Chinese University of Hong Kong, Hong Kong, China

**Keywords:** molecular dynamics simulation, metastable states, energy landscape

## Abstract

Understanding the energy landscape and the conformational dynamics is crucial for studying many biological or chemical processes, such as protein–protein interaction and RNA folding. Molecular Dynamics (MD) simulations have been a major source of dynamic structure. Although many methods were proposed for learning metastable states from MD data, some key problems are still in need of further investigation. Here, we give a brief review on recent progresses in this field, with an emphasis on some popular methods belonging to a two-step clustering framework, and hope to draw more researchers to contribute to this area.

## 1. Introduction

**Figure 1:**
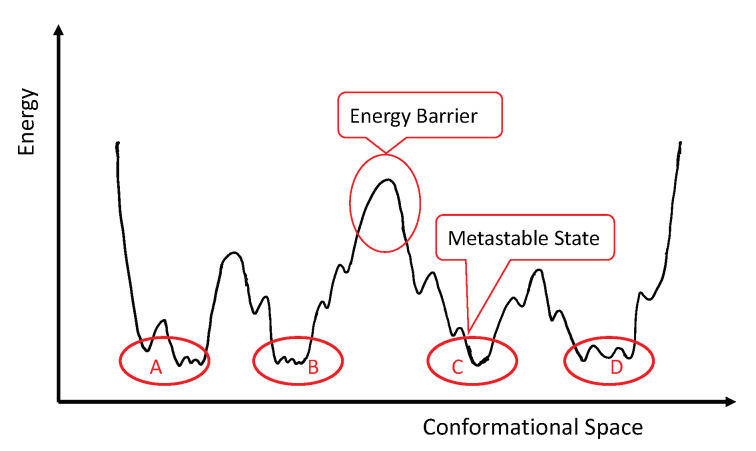
An illustration of the free energy landscape of a conformational space. There are four metastable states labeled as A, B, C and D. Conformations belonging to one metastable state, for example state B, do not easily change into conformations belonging to another metastable state, e.g., state C, due to the energy barrier between them.

Proteins are basic building blocks of life, which carry out most essential functions in a cell such as catalysation, signal transduction, gene regulation, molecular modification, etc. These capabilities depend on their three-dimensional biomolecular structures, which also undergo reversible transitions between alternative structures (also called conformations). Different conformations have different Gibbs free energy. The free energy landscape of the conformational space is rugged with a number of high-energy barriers. These barriers partition the conformational space into a set of low-energy wells, which are called metastable states. See Figure 1 for an illustration. Conformations belonging to one metastable state do not easily change into conformations belonging to another metastable state. For more details on the free energy landscape of proteins, we refer to Finkelstein and Ptitsyn [1].

The elucidation of the energy landscape and the conformational dynamics is crucial for understanding many biological processes, such as protein folding [2] and RNA folding [3], and for deciphering diseases related to improper conformational changes, such as Alzheimer’s disease, Mad cow disease, Huntington’s disease and Parkinson’s disease [4]. Several experimental methods have been proposed to study stable structures or conformational changes, such as X-ray imaging [5], nuclear magnetic resonance [6], single-molecule fluorescence resonance energy transfer [7] and Cryo-electron [8]. Computational methods have also been proposed to predict protein structure from primary sequences, such as the generative probabilistic model by Boomsma et al. [9], the sequential Monte Carlo method by Wong et al. [10], critical assessment of protein structure prediction experiment [11,12,13] and deep neural network methods [14,15,16,17], including Google’s AlphaFold [18]. However, these methods focus mainly on the protein-folding problem [19], which aims at the conformation of the lowest free energy. They cannot provide global dynamic information on conformational changes at the atomic level.

Molecular Dynamics (MD) simulations [20], which simulate conformational trajectories, have emerged and are the major source of global dynamic information at the atomic level. More specifically, MD simulations sample from a conformational space by evolving the structure based on Newton’s equations of motion. Each evolution produces a trajectory formed by a sequence of conformations at times t=0,τ,2τ,⋯,nτ, where τ denotes the observation interval. To handle the rugged energy landscape as shown in Figure 1, generalized ensemble algorithms, such as multicanonical algorithm [21] and Replica Exchange [22], are used in MD simulations to generate a wider sampling by helping the simulation trajectories pass through energy barriers with a higher probability and avoid trapping in local modes [23].

Due to the high computational cost of MD simulations, the timescale of MD trajectories is usually shorter than the typical real conformational dynamics. To bridge the timescale gap, Markov state models (MSMs) [24,25,26,27,28,29,30] were commonly used to reproduce the long-time conformational dynamics of biomolecules using MD data, see for example, Chodera and Noé [31], Wang et al. [32], and Husic and Pande [30] for a review on the status of MSMs studies. Based on MSMs, current methods for identifying metastable states from MD data mostly take a two-step clustering approach. In this review of methods for learning metastable states from MD data, we provide a detailed discussion on this two-step clustering framework, check some popular methods within this framework as well as some initiatives beyond this framework. We hope this brief review would draw more researchers to break through this two-step clustering framework for better detection of metastable states.

## 2. Learning Metastable States from MD Data

**Figure 2:**
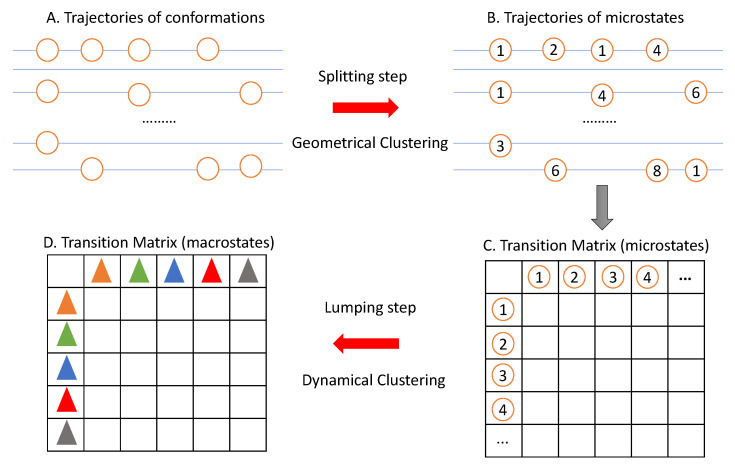
Workflow of the two-step clustering framework for learning metastable states. (**A**) Trajectories of conformations obtained from MD simulations. Each circle represents a different conformation. (**B**) Trajectories of microstates resulted from the splitting step. This step uses only the geometrical information to cluster conformations with high geometrical similarity into a microstate. Circles of the same number represent the conformations belonging to a same microstate. (**C**) The transition matrix between microstates, which counts the number of jumps between them along the trajectories. (**D**) Transition matrix between macrostates obtained from the lumping step by clustering microstates into macrostates. Each macrostate is a collection of microstates. Solid triangles with different colors represent different macrostates.

Statistically, learning metastable states from MD data estimates the distribution of conformations over the structural space. Given the molecular data (trajectories of conformations as shown in Figure 2A) from MD simulations, we want to estimate the density function $f\left(x\right)=\sum_{i=1}^{k}q_{i}f_{i}\left(x|A_{i}\right)$, where $\left\{A_{i}:i=1,2,\cdots ,k\right\}$ is a disjoint partition of the conformation space Ω, i.e., $A_{i}\cap A_{j}=\emptyset$ and $\cup_{i=1}^{k}A_{i}=\Omega$. $A_{i}$ corresponds to the basins or metastable states of the energy landscape, $q_{i}$ is the probability of conformation *x* belonging to basin $A_{i}$, and *k* is the unknown number of metastable states. Note that f(x) is a multi-mode density function. Specifically, it has *k* modes, and each $f_{i}\left(x\right)$ has one mode in its region $A_{i}$.

Taking the free energy landscape in Figure 1 for example, we may write the structural density function as $f\left(x\right)=\sum_{i=1}^{4}q_{i}f_{i}\left(x|A_{i}\right)$, with $\left(A_{1},A_{2},A_{3},A_{4}\right)$ obtained by partition the conformation space according to the energy barriers between four basins (A,B,C,D). The aim of MD data analysis is to recover basins (A,B,C,D) from data.

Before discussing the difference between estimating the structural density function and traditional density function, we shall emphasize the biological property behind the partition $\left\{A_{i}:i=1,2,\cdots ,k\right\}$ in the structural density function. Specifically, conformations belonging to the same basin (partition) shall not only have geometrical similarity at key parts but also have dynamical similarity. However, global geometrical similarity in structural space may not necessarily lead to dynamical similarity due to energy barriers. In other words, two conformations exist such that they are geometrically similar, i.e., the geometrical distance between them is smaller than some threshold; however, we may rarely observe dynamical transitions between them along the trajectories.

In the framework of traditional density estimation, the key is to get the best estimation of the density, i.e., $q_{i}$ and $f_{i}\left(x\right)$. In other words, global geometrical similarity is the only concern in traditional density estimation, and the special biological property is ignored. Thus, Bayesian sequential partition [33], which extends the idea of classification tree [34] for estimating a high-dimensional density function, can not be applied here. In the framework of Markov state model for learning metastable states, one estimates directly the partition $\left\{A_{i}:i=1,2,\cdots ,k\right\}$ and ignores $q_{i}$ and $f_{i}\left(x\right)$ because they are unimportant to metastable states.

In summary, the difficulty underlying estimating the structural density function is how to recover the partition $\left\{A_{i}:i=1,2,\cdots ,k\right\}$ and satisfy the biological property that conformations belonging to the same partition have both the geometrical and dynamical similarity. This difficulty increases with the complexity of the molecule under study. Note that there are two important parts for learning metastable states: the partition of the conformational space and the number of metastable states *k*. The metastable structure in each partition is defined as the conformation with lowest free energy.

## 3. The Two-Step Clustering Framework

A two-step clustering approach is widely used for identifying metastable states from MD data. This is due to the fact that the conformational space where MD simulations sample from is essentially a high-dimensional, continuous coordinate space. Therefore, even if the raw simulation trajectories may contain thousands of conformations, very few transitions between any specific pair of these conformations will be observed. To overcome this sparsity problem at the conformation level, a two-step clustering framework, a clever idea, is commonly adopted for analyzing the trajectories of conformations. See Figure 2 for an overview of this two-step clustering framework.

Firstly, a splitting step is used to group conformations into a number of microstates according to their structural (geometric) similarity. In this step, we introduce a new concept, called microstate, which is defined as a set of conformations with high geometrical similarity. Actually, conformations belonging to the same microstates are assumed to have both geometrical similarity and dynamical (kinetic) similarity, which ensures the fast converting among them. It is expected to observe more transitions between microstates than between conformations; thus, we can hopefully get a statistically stable transition matrix between microstates. Since this step uses only the geometric information of conformations, we refer it to as the geometric clustering step.

Secondly, a lumping step is used to cluster further microstates into macrostates (also called metastable states) based on the transition matrix between microstates. Thus, a macrostate (metastable state) is as a set of microstates with high dynamical similarity. This step depends on the dynamic information between microstates; thus, it is referred to as the dynamical clustering step.

To have relatively stable jumps between microstates, the number of microstates should be selected carefully. If it is too large, the transition frequency between microstates will be very low. If it is too small, a microstate may contain conformations that are separated by energy barriers. Both situations will prevent the detection of true metastable states. In addition, ignoring the geometrical information in the lumping step is problematic and gives undesired results. In the following, we dive into details of this two-step clustering framework.

### 3.1. The Splitting Step: Geometrical Clustering

The splitting step corresponds to the transition from Figure 2A to Figure 2B. The input of this step is the vector data in $R^{m}$ of *n* samples, where *n* is the total number of conformations in all trajectories, and *m* is the dimension of the molecule, depending on the pre-processing of the MD data. A conformation can be represented by the coordinates of all atoms or its torsion angles. Thus, the dimension *m* of these two different representations may be different. K-means [35] and K-medoids [36] algorithms are widely used in this step due to their easy implementation.

To improve the efficiency of these two algorithms, dimension reduction methods such as principle component analysis are applied before geometrical clustering. The principle components (PCs) of coordinates and that of torsion angles are commonly used as the representation of a conformation, see for example Mu et al. [37], Altis et al. [38]. For more information about principle component analysis (PCA) of molecular dynamics, we refer to Sittel et al. [39], where a detailed comparison of PCA on the use of Cartesian and internal coordinates is given.

The main concerns about K-means/K-medoids algorithms are as follows. Firstly, both of them give a local optimum instead of a global optimum due to the large sample size. This means there may be some conformations belonging to the same microstates that are clustered into different microstates, and thus leads to bad basins. Researchers usually try to run the K-means or K-medoids algorithm multiple times to get better results. Second, the aim of K-means/K-medoids algorithms, essentially, is to obtain an η-cover of the vector space with centers $\left\{P_{1},P_{2},....P_{k}\right\}$ such that for each conformation P, there is an $P_{i}$, such that $d(P,P_{i})\leq \eta$, where d(·,·) is a distance function defined for any two vectors, and η can be understood as the similarity threshold for defining microstates. It is impossible for us to find a suitable η and *k* in real applications, which implies the inevitable difficulty of K-means/K-medoids algorithms in the splitting step to give satisfactory microstates.

In principle, any clustering algorithm (see Jain [40] for a review) taking vector data as input can be used for geometrical clustering. The computational burden and the quality of resultant microstates are the main concerns in this step. In addition, there are methods proposed to improve the quality of microstates from this step. We will discuss them in Section 3.3.

### 3.2. The Lumping Step: Dynamical Clustering

The lumping step corresponds to the transition from Figure 2C to Figure 2D. The input of this step is the transition matrix between microstates obtained from the splitting step. There are many different strategies for this dynamical clustering. We introduce below the representative ones and discuss their performance on an MD alanine dipeptide dataset, a well-understood molecule with six metastable states. The reason for choosing this historic dataset is that we know the ground truth of its metastable states, which is crucial for us to understand the performance of each method.

**Figure 3:**
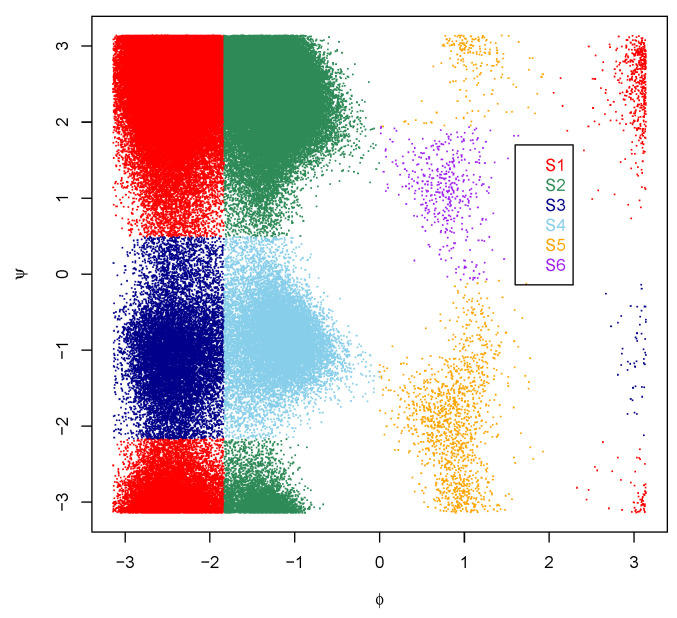
The scatter plot of ϕ-ψ of the alanine dipeptide with ϕ,ψ∈[−π,π]. The partition of ϕ-ψ space into six clusters follows that given in Chodera et al. [41].

The MD trajectory data are taken from Chodera et al. [41], which consists of 974 20-ps NVE simulations with conformations stored every 0.1 ps, and there are 194,800 conformations in the dataset. The detailed simulation information can be found in Chodera et al. [41]. The conformation space of the alanine dipeptide can be represented by two torsion angles ϕ and ψ [41]; thus, it is a simple molecule often taken as a benchmark. Figure 3 shows the scatter plot of ϕ-ψ of these 194,800 conformations with transition matrixes between its six metastable states, shown in Table 1, where the partition of the conformational space into six clusters (metastable states) follows that given in Chodera et al. [41]. Note that these six metastable states are given by a manual partition according to the estimated landscape from the parallel tempering simulation [41]. To eliminate the impact of microstates from the splitting step on the lumping step, the microstates of the alanine dipeptide are obtained by a grid method that partitions the ϕ-ψ space into 80×80 grids and takes each non-empty grid as a microstate.

*Perron Cluster Cluster Analysis (PCCA, Deuflhard et al. [42]) and Its Variants*. PCCA is based on two important observations of transition matrix *P* between microstates: (P1) if *P* has an *s* block-diagonal structure, its eigenvalue λ=1 is *s*-fold, which is used to identify the number of macrostates; (P2) the sign structure of the eigenvector corresponds to the assignment of macrostates. Thus, the idea of PCCA is mathematically solid and easy to implement. In practice, one should input the number of clusters (metastable states) generated from PCCA, which is difficult to estimate for real applications.

**Figure 4:**
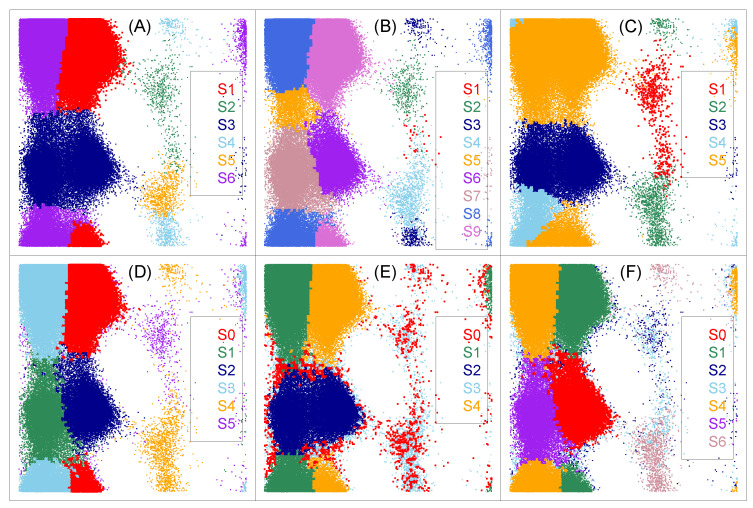
Clustering results of the alanine dipeptide from PCCA, PCCA+ and Gib algorithms. The axis is the same as that in Figure 3. (**A**) PCCA with 6 clusters; (**B**) PCCA+ with estimated number of cluster belonging to [3, 9]; (**C**) PCCA+ with estimated number of cluster belonging to [5, 7]; (**D**) GSA with 6 clusters; (**E**) GSA with 5 clusters; (**F**) GSA with 7 clusters.

In PCCA, the true transition matrix between microstates is assumed to be block-diagonal, i.e., $D=\mathrm{diag}(D_{11},D_{22}$, $\cdots ,D_{kk})$, where *k* is the number of macrostates, and the observed transition matrix P=D+E, where *E* is the perturbation matrix representing error of the observations. This assumption may fail sometimes. Consider a special case where microstates are macrostates, we find that the true transition matrix can not be block-diagonal (see Table 1 for example). Secondly, PCCA can be understood as finding a macrostate assignment, based on property (P1) and (P2), by maximizing the sum of diagonals of the transition matrix between macrostates, i.e., the metastability of macrostates. However, this kind of object may not directly lead to macrostates with the biological property that conformations belonging to the same partition have both the geometrical and dynamical similarity. That is, although conformations within the same macrostates obtained by PCCA have high dynamical similarity, the geometric similarity between conformations belonging to the same macrostates are not guaranteed. This is partly due to its ignorance of geometric information when clustering microstates into macrostates. To make this point clear, we show in Figure 4A the clustering results of alanine dipeptide from PCCA by setting the number of clusters (metastable states) as its true number of clusters 6, which is very different from the reference clustering labels shown in Figure 3. Typically, metastable states S3 and S4 in Figure 3 are recognized as one metastable state in Figure 4A. However, PCCA gives satisfied results according to the corresponding transition matrix given in Table 2, which has a sum of diagonals close to that of the true transition matrix in Table 1. These facts together imply that the mathematical optimal solution provided by PCCA may not be biologically meaningful.

Different versions of PCCA, such as PCCA+ [43] and Flux PCCA (FPCCA, Beauchamp et al. [44]), are proposed to improve its robustness to random perturbations. PCCA+ needs users to give the range of number of clusters (metastable states). Figure 4B,C shows the clustering results of the alanine dipeptide from PCCA+ with different ranges, and the results are undesired.

*Gibbs Sampling Algorithm (GSA, Wang et al. [45])*. GSA is based on a Poisson model assuming that the observed number of jumps between macrostates follows a Poisson distribution. Taking the transition matrix between macrostates as a parameter, given this Poisson model, we have the likelihood function for the macrostates assignment of microstates. The transition matrix and the macrostates assignment of microstates is learned by maximizing the likelihood function. To the best of our knowledge, this is the first attempt on statistical modeling of the transition matrix between macrostates. The major point of GSA is to take the unknown number of macrostates as input. Figure 4D–F shows the clustering results of alanine dipeptide from GSA with different numbers of clusters. When users specify the right number of clusters, as shown in Figure 4D, GSA gives quite good results. However, when users specify a bad one, the results will be bad, as shown in Figure 4E,F.

**Figure 5:**
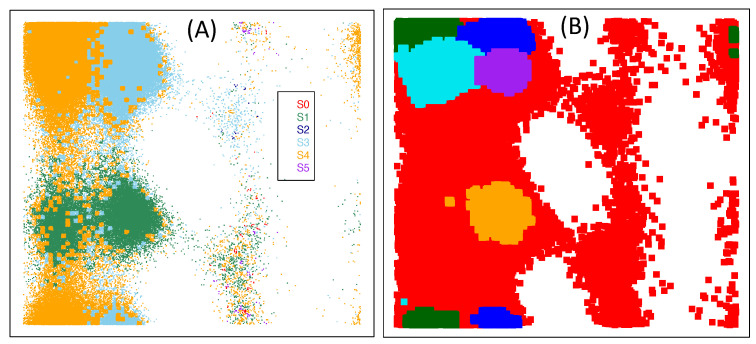
Clustering results of the alanine dipeptide from MPP (**A**) and MVCA (**B**). The axis is the same as that in Figure 3. Different colors in the figure present different clusters.

*Most Probable Pathway (MPP, Jain and Stock [46])*. The idea underlying MMP is straightforward: it merges the microstate with its neighboring microstates on its most probable pathway that has the lowest free energy. The merit of MPP is that it does not require an estimated value or range on number of clusters (metastable states) as input. However, due to the discreteness of MD data, there exits undesired cases; for a microstate belonging to state A, we may observe its most probable pathway leads to a microstate with the lowest free energy belonging to state B. According to the principle of MPP, we should merge this microstate into state B, which is undesired. To make this point clear, we show in Figure 5A the clustering results of alanine dipeptide from MPP. As shown in the figure, some microstates belonging to state S4 are clustered wrongly into states S1 and S3.

*Minimum Variance Clustering Approach (MVCA, [47])*. Husic et al. [47] considered a different strategy in the second step by using symmetric Jensen–Shannon divergence to measure the similarity between microstates and Ward’s minimum variance criterion to do the agglomerative clustering. Following this idea, one may use other distance metrics to measure the similarity between microstates, and use agglomerative clustering to cluster microstates into macrostates. We show in Figure 5B the clustering results of the alanine dipeptide from MVCA.

In summary, we discussed the performance of different strategies for the lumping step by providing the same (and almost ideal) microstates to different methods. That is, the performance of each method only depends on the strategy for the lumping step. According to results shown in Figure 4 and Figure 5, the performance PCCA(+), MPP, MVCA and GSA depends strongly on the number of macrostates from the algorithm. Importantly, the scatterplots from PCCA(+), MPP and MVCA are different from the ground truth shown in Figure 3, although their underlying principle is well-understood. This may be partially caused by the fact that the lumping step uses only the dynamical information between microstates but ignores the geometrical information between them. GSA shows good performance when microstates are well defined and the number of macrostates is correctly specified, which are quite difficult as discussed before.

### 3.3. Refinements to The Framework

Researchers have noticed two shortcomings for the above two-step procedure [25,48,49] when applied to complex systems: (1) the quality of microstates is not guaranteed, which has a strong impact on the downstream analysis; (2) some poorly sampled states may dominate the coarse-grained model. In the following, we discuss a few strategies to refine the two-step framework.

*Iterative framework to improve microstates*. An iterative framework [48,49] was proposed to obtain better microstates. It goes as follows: (a) splitting macrostates by geometrical clustering using stepwise K-means algorithm by incorporating other information, such as escape probability; (b) lumping microstates into macrostates by dynamical clustering using PCCA, PCCA+ or any other method; (c) repeating (a–b) until it converges. Note that there is only one macrostate in the fist iteration. Essentially, this kind of iterative algorithm is to improve the quality of microstates. In other words, if we have another way to get better microstates, this iterative framework may not be helpful in basin estimation. This iterative method may improve the quality of microstates, but it still can not make sure the resultant microstates are good enough.

*Other methods for the splitting step.* Ignoring the dynamic information between conformations, geometric clustering is just a clustering problem with vectors as input. Based on this observation, all other methods for vector clustering [40] are applicable to the current problem. For example, Sittel and Stock [50] and Liu et al. [51] proposed a density-based clustering [52,53] method to cluster conformations into microsates and then used MPP or PCCA to further cluster microstates into macrostates. Taking a density-based clustering method in the splitting step avoids the difficulty of local convergence from K-means or K-medoids algorithms. However, selecting the bandwidth, the key parameter determining the number of clusters, for density-based clustering is still in need of further investigation. Furthermore, dimension reduction methods other than PCA are suggested to extract important coordinates before going to the splitting step, see Sittel and Stock [54] for a discussion.

*Reducing the impact of poorly sampled microstates*. Bayesian agglomerative clustering engine (BACE, Bowman [55] and Hierarchical Nyströn expansion graph (HNEG, Yao et al. [56] are proposed to amend the shortcoming of PCCA and PCCA+, that they tend to identify poorly sampled states as being kinetically distinct from their neighbors [25]. Specifically, BACE identifies coarse-grained states by finding sets of states that have the same kinetics (i.e., transition probabilities to other states) within statistical uncertainty through Bayes factor. See Bowman [55] for details. HNEG attempts to solve this problem by placing more emphasis on well-sampled states than poorly sampled ones based on the idea that the whole transition matrix can be approximated by a sub-stable transition matrix between well-sampled states. The metastable macrostates are obtained by applying PCCA (PCCA+) to the sub-stable transition matrix. In other words, HNEG is an improved version of PCCA and PCCA+. We refer to Bowman et al. [57] for a review and a comparison on the performance of some of these methods, where the authors pointed out PCCA (PCCA+) has a similar performance with BACE and HNEG, but it is better than MPP.

For complex systems, the shortcomings of the lumping step discussed before still exist. The additional difficulty comes from the splitting step, which, as discussed in Section 3.1, is from the following two fundamental facts: (1) K-means/K-medoids algorithms are very difficult to converge, and (2) the number of microstates from them is also very difficult to determine. Although different methods are proposed to improve the quality of microstates, we still do not know whether they are good enough for downstream analysis on complex systems, as these methods work in an intuitive way. Thus, we expect new ideas to overcome these limitations. The optimal reaction coordinates [58] that treat the free energy as a function of reaction coordinates is a good example on this direction.

## 4. Some Extensions

In previous sections, we gave a brief review on methods under the two-step clustering framework for learning metastable states. Here, we discuss some extensions for modeling MD data beyond MSMs.

*Deep neural networks (DNN) for learning molecular dynamics.* DNN has rapidly developed in recent years due to its successful application to image processing. See LeCun et al. [59] for a review. Researchers are motivated to apply DNN to other areas including predicting protein folding and exploring the landscape of proteins. For example, Wu et al. [60] and Mardt et al. [17] proposed a deep generative Markov state model based on deep neural network to learn molecular dynamics and sample conformations from conformation space. The key elements in their DNN models are (1) a DNN encoding the coordinates information into latent space, (2) a Markov transition model between elements in latent space and (3) a generative model decoding from latent space to coordinates information. These DNN models are promising. However, to train the DNN, we should know the number of macrostates first, which is unknown to us. How to learn automatically the number of macrostates from MD data is still open.

DNN is a powerful tool for many problems, especially for image processing. However, a lack of explanation of results from deep learning is the key point that hinders its application on other areas, for example, biology. It is well known that obtaining MD data is time-consuming, and it is helpful to get it from deep learning, which is potential direction for future works. For more discussions on machine learning methods for MD data analysis, we refer to Noé [61].

*Improvements on MSMs.* Markov state models assume a Markov chain on a discretization of the state space. However, it is difficult to apply to high-dimensional biomolecular systems. The quality and reproducibility of MSMs are therefore limited. Differently, projected Markov Models (PMMs, Noé et al. [62]) only assume that the full phase space molecular dynamics is Markovian, and a projection of this full dynamics is observed on the discrete states. However, estimating PMMs is very difficult. In addition, Dynamic Graphical Models (DGMs, Olsson and Noé [63]) are proposed to deal with the case where the size of global metastable states grow exponentially with the system size. Similar to how spins interact in the Ising model, DGMs describe molecules as assemblies of coupled subsystems, and the change of each subsystem state is only governed by the states of itself and its neighbors. We refer to their original paper for more details about PMMs and DGMs.

## 5. Discussion and Outlook

In this paper, we reviewed some popular methods for learning metastable states from molecular dynamics data, and most of them belong to a two-step clustering framework including a splitting step and a lumping step. The performance of popular methods is illustrated based on MD data of the alanine dipeptide.

In the splitting step, one wants to obtain microstates by clustering conformations into microstates while ignoring the dynamical information underlying them. K-means and K-medoids are commonly used in this step. However, they suffer from drawbacks such as being stuck in a local mode and not easily obtaining microstates with biological properties that conformations belonging to the same partition have both the geometrical and dynamical similarity. Density-based clustering is used to avoid the drawback of these two methods, but it introduces a new difficulty on selecting the threshold to define the local density. Microstates from the splitting step have a strong impact on the downstream analysis. Bad microstates may lead to bad metastable states. For simple systems such as alanine dipeptide, a grid clustering method is available for geometrical clustering to get better microstates. However, for proteins such as HP35 NLE/NLE, methods such as MPP will inevitably give poor results due to bad microstates.

In the lumping step, there are many methods proposed for dynamical clustering. Each method has its own philosophy. PCCA has solid mathematical foundation, the idea behind MPP is straightforward, and GSA is based on a Poisson model. The key problem underlying them is their failures on estimating the number of metastable states; however, PCCA and GSA should take this unknown number as input. In addition, they strongly rely on the microstates from the splitting step. Bad microstates inevitably lead to bad macrostates no matter what method is used in the lumping step.

The key features of this two-step framework are (1) the quality of microstates has a strong impact on the quality of macrostates and (2) a separate consideration of geometric clustering and dynamical clustering. That is, the geometric information of conformations is only used in geometric clustering, and dynamical clustering uses only the dynamic information. Although an iterative framework is proposed to refine the microstates, we still can not make sure the conformations belong to the same microstates from the splitting step with the biological property. Thus, further investigation should focus on how to obtain high-quality microstates and how to combine the geometric and dynamic information to learn the metastable states.

Deep neural networks (DNN) are proposed to learn molecular dynamics from MD data, and thus learn the metastable states. However, it can not learn the number of metastable states. Instead, it should take this number as input. In other words, how to design a DNN for learning automatically the number of metastable states is still open. How to verify the DNN learned from MD data also needs further study.

Another interesting problem related to metastable states learning is to explore the relationship between local geometric similarity and dynamical similarity. We believe that the dynamic similarity between conformations comes from the local similarity in geometry. The advantage of DNN is to learn this relationship automatically from MD data, which is hidden from us and loses its interpretation. However, the two-step clustering framework ignores this point.

Finally, all of these methods do not take into consideration the statistical uncertainty of the MD data. There is great need of a full statistical model for MD data. GSA is a statistical model for the transition matrix between macrostates, and it has a good performance when we know the true numbers of macrostates and microsates are well defined. This fact sheds some light on statistical modeling on MD data. Based on the success of DNN, we should incorporate dimension reduction/variable selection into the statistical model for MD data, which would enable us to infer metastable states and the number of macrostates in a full statistical manner.

## Figures and Tables

**Table 1:** Transition matrix of the benchmark clusters of the alanine dipeptide with S1–S6 shown in Figure 3.

	S1	S2	S3	S4	S5	S6
S1	0.9457	0.0477	0.0062	0.0004	0.0000	0.0000
S2	0.0609	0.9365	0.0004	0.0021	0.0000	0.0002
S3	0.0403	0.0021	0.8939	0.0636	0.0000	0.0000
S4	0.0020	0.0090	0.0526	0.9356	0.0008	0.0000
S5	0.0013	0.0013	0.0000	0.0098	0.9718	0.0158
S6	0.0000	0.0401	0.0000	0.0000	0.0519	0.9080
Sum of diagonals: 5.591479						
Mean of diagonals: 0.9319131						
Minimal of diagonals: 0.8939						

**Table 2:** Transition matrix between clusters of the alanine dipeptide obtained by PCCA with S1–S6 shown in Figure 4A.

	S1	S2	S3	S4	S5	S6
S1	0.9352	0.0003	0.0018	0.0000	0.0000	0.0626
S2	0.0477	0.9131	0.0000	0.0068	0.0324	0.0000
S3	0.0042	0.0000	0.9752	0.0000	0.0004	0.0202
S4	0.0000	0.0032	0.0000	0.9104	0.0816	0.0048
S5	0.0000	0.0269	0.0175	0.0672	0.8884	0.0000
S6	0.0508	0.0000	0.0068	0.0000	0.0000	0.9424
Sum of diagonals: 5.564797						
Mean of diagonals: 0.9274662						
Minimal of diagonals: 0.8884						
